# A Panel of Stably Expressed Reference Genes for Real-Time qPCR Gene Expression Studies of Mallards (*Anas platyrhynchos*)

**DOI:** 10.1371/journal.pone.0149454

**Published:** 2016-02-17

**Authors:** Joanne R. Chapman, Anu S. Helin, Michelle Wille, Clara Atterby, Josef D. Järhult, Jimmy S. Fridlund, Jonas Waldenström

**Affiliations:** 1 Centre for Ecology and Evolution in Microbial Model Systems, Linnaeus University, Kalmar, Sweden; 2 Zoonosis Science Center, Department of Medical Biochemistry and Microbiology, Uppsala University, Uppsala, Sweden; 3 Section for Infectious Diseases, Department of Medical Sciences, Uppsala University, Uppsala, Sweden; University of Lleida, SPAIN

## Abstract

Determining which reference genes have the highest stability, and are therefore appropriate for normalising data, is a crucial step in the design of real-time quantitative PCR (qPCR) gene expression studies. This is particularly warranted in non-model and ecologically important species for which appropriate reference genes are lacking, such as the mallard—a key reservoir of many diseases with relevance for human and livestock health. Previous studies assessing gene expression changes as a consequence of infection in mallards have nearly universally used β-actin and/or GAPDH as reference genes without confirming their suitability as normalisers. The use of reference genes at random, without regard for stability of expression across treatment groups, can result in erroneous interpretation of data. Here, eleven putative reference genes for use in gene expression studies of the mallard were evaluated, across six different tissues, using a low pathogenic avian influenza A virus infection model. Tissue type influenced the selection of reference genes, whereby different genes were stable in blood, spleen, lung, gastrointestinal tract and colon. β-actin and GAPDH generally displayed low stability and are therefore inappropriate reference genes in many cases. The use of different algorithms (GeNorm and NormFinder) affected stability rankings, but for both algorithms it was possible to find a combination of two stable reference genes with which to normalise qPCR data in mallards. These results highlight the importance of validating the choice of normalising reference genes before conducting gene expression studies in ducks. The fact that nearly all previous studies of the influence of pathogen infection on mallard gene expression have used a single, non-validated reference gene is problematic. The toolkit of putative reference genes provided here offers a solid foundation for future studies of gene expression in mallards and other waterfowl.

## Background

Measuring host cellular responses to pathogens is a key requirement to understand pathogenesis and disease progression [1–3]. One approach is to measure changes in mRNA transcription of genes of interests in infected versus uninfected individuals via real-time quantitative PCR (hereafter qPCR). Such studies allow elucidation of the contribution of individual genes and, in some cases, genetic pathways involved in host immune responses to pathogens (e.g. [4–6]). By measuring gene expression over a time-course of infection, it is possible to determine the speed, magnitude and longevity of the immune response (e.g. [7–9]). It is especially important to quantify such information in species which act as reservoirs of pathogens of import to humans, such as zoonoses. The qPCR approach to measure gene expression can be particularly useful for species where large-scale multiplex methods such as microarrays, Serial Analysis of Gene Expression (SAGE) and RNA seq are not viable.

One such species is the mallard (*Anas platyrhynchos*); the most widely distributed [10, 11] and intensively farmed [12] duck species in the world. It is a key reservoir for many diseases, including economically important zoonotic pathogens such as *Salmonella* spp., *Campylobacter* spp. and avian influenza virus (AIV). Mallards have been subject to intensive research to understand host immune responses to zoonotic pathogens, including qPCR studies assessing changes in gene expression after infection (e.g. [13–16]). However, to date only a single study has assessed the stability of potential reference genes (RGs) in mallards [17], and then only three RGs were considered: glyceraldehyde-3-phospahte dehydrogenase (GAPDH), β-actin (ACTB) and 18S rRNA. The authors concluded that 18S rRNA was a suitable single RG for normalisation of qPCR data in ducks [17]. However, there are numerous issues with using 18S rRNA to normalise data (reviewed in [18]), and the use of a single RG for normalisation is not recommended [19, 20].

The fundamental requirement for an RG is that it displays stable expression across the different treatment groups under consideration. When measuring individual responses to disease, this means that the RG must not itself be up- or down-regulated in response to infection. This can be tested empirically by measuring the cycle threshold (Ct, also known as the quantification cycle, Cq) values of infected and uninfected individuals for a panel of potential RGs, and determining whether expression levels are uniform across groups, for example via the use of the software programmes BestKeeper [21], geNorm [20] and/or NormFinder [22]. Additionally, the RG(s) should have similar Ct values as the gene(s) of interest (GOI(s)), and enough RGs need to be used to reach a threshold stability value [18–20].

Here, we provide a panel of eleven putative reference genes for the mallard. We show that different putative RGs are stable in different tissues, but that for each tissue analysed it is possible to find a combination of two RGs that provide adequate normalisation when investigating low pathogenic AIV infected versus control (uninfected) ducks. This panel of potential RGs is of general applicability to all future studies of gene expression in mallard, and is not limited to those investigating the consequences of infection.

## Materials and Methods

### Animal experiments

Thirty three male mallards were obtained from a commercial breeding facility one day post hatch and transported to a biosecurity level two (BSL2) animal facility at the Swedish National Veterinary Institute (SVA). Ducks were housed indoors at SVA with a 12 hour day-night cycle and had access to a pool for swimming, food and water (*ad libitum*). At the start of the experiment, ducks (9 weeks of age) were moved to HEPA filtered rooms with positive air pressure and double doors. Cloacal swabs and blood samples (brachial vein) were taken from all individuals at this time to confirm they were AIV negative via qPCR and ELISA, further details are provided as supporting information (S1 Appendix).

All animal experiments were conducted in accordance with regulations provided by the Swedish Board of Agriculture and were approved by the Ethical Committee on Animal Experiments in Uppsala (permit number C63/13). Animals were monitored daily for signs of disease or stress. We observed no such signs in any individual, and all individuals remained alive and healthy until their pre-determined endpoint. A protocol was in place for humane euthanasia of severely ill animals, but was not required during our experiment. Low pathogenic avian influenza (LPAI), as used here, is known to produce few, if any, signs of infection in mallards [23] and this was borne out in our experiment, whereby we observed no morbidity or distress in infected individuals.

### AIV infection

Three days prior to the start of the experiment, ducks were randomly divided into three treatment groups (uninfected controls, *n* = 5; experimentally infected, *n* = 3; and contact infected, *n* = 25), with each group being placed in a separate room. The experimental set-up is depicted in S1 Fig.

We used a semi-natural infection technique, in an effort to mimic natural conditions with respect to viral transmission, viral load and passage through the host. To do this, three ducks were artificially infected with LPAI A/Mallard/Sweden/51833/2006 (H1N1) via oesophageal inoculation three days (t-3) before the start of the experiment, as described in Järhult *et al*. [24]. At the start of the experiment (t0), 25 uninfected ducks were moved to a room containing the three artificially inoculated ducks (S1 Fig). All individuals could interact freely and, in addition to a water drinker, a 170-liter pool was provided for swimming and drinking, facilitating efficient natural transmission from inoculated to uninfected ducks (hereafter termed contact-infected ducks). Five ducks housed in a separate room were kept AIV-free throughout the experiment to serve as uninfected controls. AIV infection status of all individuals was confirmed daily via qPCR following the methods of Tolf *et al*. [25], further details are provided as supporting information (S1 Appendix).

### Tissue collection and RNA extraction

At specific time points (t = 0.5, 1, 2, 4 and 7 days after introduction of the inoculated ducks, hereafter days post infection, dpi), five of the contact-infected ducks were randomly selected and sequentially euthanized humanely by mechanical disruption of the brainstem via the use of a CASH Poultry Killer (Accles & Shelvoke, Sutton Coldfield, UK). The five control ducks and three experimentally inoculated ducks were sequentially euthanized on day three, equivalent to 6 dpi for the inoculated ducks. Necropsies were performed immediately after death and the following tissues collected: blood; lung; spleen; two sections of the gastrointestinal tract (GI) corresponding to the distal jejunum, anterior to Meckel’s diverticulum (hereafter GI2), and the distal ileum, anterior to the joining of the caecum (hereafter GI4); and colon. Approximately 150 mg of each tissue was sliced into ~2 mm^3^ pieces, immediately snap frozen in liquid nitrogen and thereafter stored at -80°C until required for analysis.

The sampling strategy outlined above and in S1 Fig was part of a larger experiment analysing the effects of LPAI infection on gene expression in mallards (unpublished data). As a first step, it was important to identify stable RGs. Thus, a subset of representative samples across treatment groups were chosen, as recommended in the QBase+ manual (https://www.biogazelle.com/sites/default/files/public_file/qbaseplus_manual.pdf). We selected 13–14 individuals comprising all uninfected ducks for which a sample was available (*n* = 4–5) and one third of the infected ducks (*n* = 9) spread across the time points (see S1 Table for samples used). Only four uninfected samples were available for GI2 and GI4 due to labels peeling off tubes during storage in liquid nitrogen. RNA was extracted from the selected samples per tissue, using the RiboPure Kit (Ambion) following the manufacturers’ protocol, followed by DNase treatment. To confirm that DNase treatment successfully removed contaminating genomic DNA from RNA extracts, intronic primers were used to confirm absence of PCR amplification. Additionally, treated extracts were run on an agarose gel containing 2% bleach to visualise quality and integrity of RNA and confirm absence of DNA. Further details are provided as supporting information (S1 Appendix).

### Reverse transcription and qPCR

cDNA was synthesised and amplified in a two-step process. Synthesis (step one) was performed via reverse transcription (RT), at a standard tissue-specific concentration, using Superscript III (Invitrogen, Carlsbad, CA) and random hexamers (Invitrogen). Further details are provided as supporting information (S1 Appendix). cDNA was then amplified (step two) via qPCR to determine the transcription level of candidate RGs. Each reaction consisted of 5 μl 1:10 diluted cDNA, 10 μl iQ^™^ SYBR Green Supermix (BioRad, Carlsbad, CA), 150 mM each of the forward and reverse primers and DNase free H2O to a final volume of 20 μl. Negative controls, comprising cDNA-free reactions and RT-minus reactions, were included for every gene and tissue combination (see also S1 Appendix). qPCR reactions were run on a LightCycler480 (Applied Biosytems, Foster City, CA) with the following cycle for all genes: an initial denaturation of 95°C for three minutes, followed by 45 cycles of 95°C for 10 seconds (s), and 58°C for 30 s, with data acquisition at the end of each elongation step. Immediately following qPCR, a melt analysis was performed (95°C for 10 s with a ramp rate of 4.4°C/s, 58°C for one min with a ramp rate of 2.2°C/s, followed by a slow incremental increase in temperature to 97°C with a ramp rate of 0.11°C/s and continual acquisition), finally samples were cooled to 40°C for 30 s. All qPCR reactions were run in duplicate.

Primers (Table 1) were designed, with reference to the duck genome [26], using the primer design tool in NCBI (http://www.ncbi.nlm.nih.gov/tools/primer-blast/). To confirm amplification of the desired products, 2–5 PCR products per gene were sequenced (Eurofins MWG Operon, Elmsburg, Germany) and blasted (Nucleotide BLAST, NCBI) to confirm homology with the expected genes. Our choice of candidate RGs to test was informed in part by those used previously in chicken [27, 28], as well as discussions with colleagues with experience in gene expression analysis.

**Table 1:** Primers used in this study. F denotes the forward primer and R the reverse primer. Annealing temperature (Ta) expressed in °C and length in base pairs (bp).

Gene Symbol	Gene Name		Primers	Ta	Length
ACTB	β-actin	F	5’-CCGTAAGGACCTGTACGCCAACAC-3’	60	208
		R	5’-GCTGATCCACATCTGCTGGAAGG-3’		
ALB	Albumin	F	5’-ACCAGACTTCGTTCAACCGT-3’	60	146
		R	5’-CACCAAGGATTGTAGGGGCA-3’		
GAPDH	Glyceraldehyde-3- phosphate dehydrogenase	F	5’-GGTTGTCTCCTGCGACTTCA-3’	60	164
		R	5’-TCCTTGGATGCCATGTGGAC-3’		
HPRT	Hypoxanthine guanine phosphoribosyl transferase	F	5’-GTCCTGTCCATGATGAGCCC-3’	60	161
		R	5’-ACCACTTGCGCAACCAAAAA-3’		
HSP90	Heat shock protein 90	F	5’-AGGTTGTTGTGTCCAACCGT-3’	60	150
		R	5’-CCAGGTGTTTCTTTGCTGCC-3’		
NDUFA	NADH dehydrogenase 1 alpha subcomplex	F	5’-CCTGAAGTTCAACAAGGGGC-3’	59	123
		R	5’-TCTGGAACGAAGTGAGGGAC-3’		
RPL4	Ribosomal protein L4	F	5’-CCTGGGCCTTAGCTGTAACC-3’	60	115
		R	5’-AAGCTGAACCCATACGCCAA-3’		
RPL30	Ribosomal protein L30	F	5’-CTCAATGTTGTTGCCGCTGT-3’	60	119
		R	5’-GCAAAGCCAAGCTGGTCATC-3’		
RPS13	Ribosomal protein S13	F	5’-AAGAAAGGCCTGACTCCCTC-3’	59	82
		R	5’-TGCCAGTAACAAAGCGAACC-3’		
SDHA	Succinate dehydrogenase complex, subunit A	F	5’-GACACAGTGAAAGGCTCCGA-3’	60	90
		R	5’-CTCCAGCTCTATCACGGCAG-3’		
UBE20	Ubiquitin-conjugating enzyme E2O	F	5’-AGCATCCCCCTTTCCATCAA-3’	59	91
		R	5’-CAACCCTGTCTCCTGGCTTA-3’		

### Statistical analyses

The algorithms GeNorm [20] and NormFinder [22] were used to quantify stability of the eleven putative RGs. GeNorm calculates an internal control gene stability measure M for each gene (M value), by computing the average pairwise variation of a particular gene with all others under consideration [20]. Genes with the lowest M values have the most stable expression, with values under 0.5 indicative of acceptably stable expression. GeNorm ranks genes from least to most stable by stepwise exclusion of the least stable gene and repetition of the analysis until only two most stable genes remain. Furthermore, GeNorm calculates the number of genes required for accurate normalisation (V value) by comparing the coefficient of variation of the *n* most stable gene(s) with the *n*+1 most stable genes (*V*_*n/n+1*_) whereby a V value below 0.15 indicates that *n* genes are sufficient for stabilisation [20]. We implemented GeNorm analyses in QBase+ [29]. NormFinder calculates an internal stability value for each gene based on expression variance, and also provides the option to consider variance between experimental subgroups [22], which we set as the variance in expression between infected and uninfected individuals. NormFinder requires input data to be linearized, which we did by transforming raw Ct values using the formula RQ = 1 / (2^(Ct,sample−Ct,min)^) where RQ is the relative quantity, Ct,min is the lowest Ct value obtained for any sample for that gene and tissue combination, and Ct,sample is the Ct value of the sample being standardised [30]. We implemented NormFinder analyses in Excel using a freely available macro (http://moma.dk/normfinder-software). All data was plotted in GraphPad Prism v.6.03 (GraphPad Software Inc, San Diego, CA).

## Results

### Candidate RG expression levels

**Fig 1:**
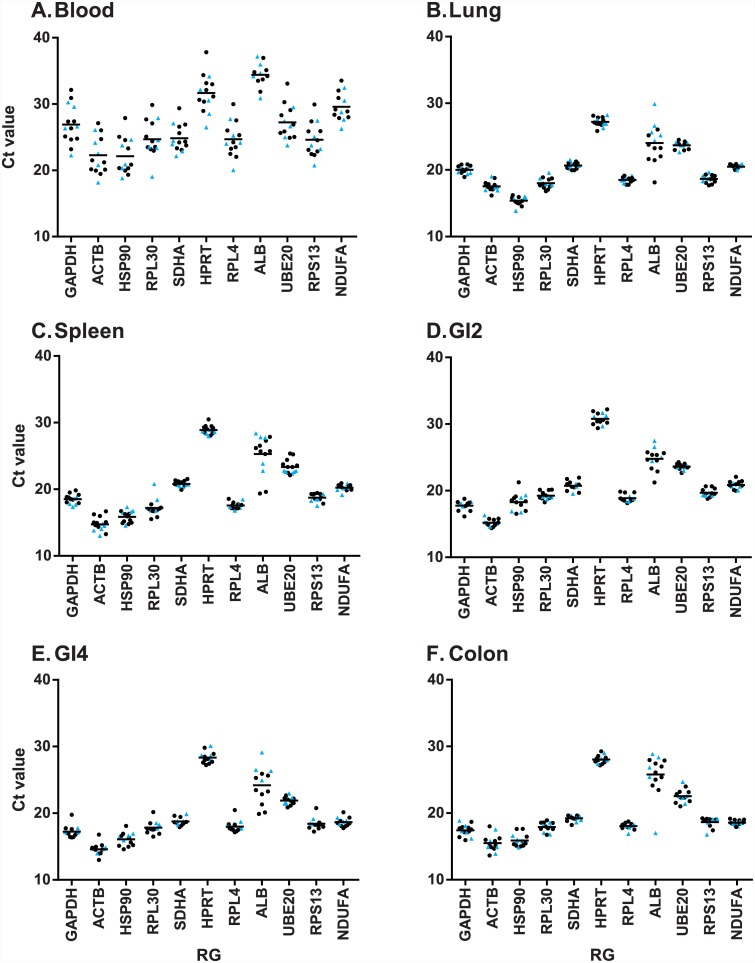
Expression levels of each putative RG per tissue. Expression level expressed in terms of Ct value whereby a lower Ct value represents higher expression, of the eleven putative reference genes (RGs) across six mallard tissues. (A) Blood. (B) Spleen. (C) Lung. (D) GI 2 (distal jejunum). (E) GI 4 (distal ileum). (F) Colon. Uninfected individuals are shown with blue triangles, infected individuals with black circles.

HPRT had the lowest expression levels in all tissues except blood, while ACTB had the highest expression levels in all tissues except lung (Fig 1). With two exceptions, most genes had tightly clustered expression levels within each tissue type. The first exception was blood samples, for which there was notably higher variance in expression levels amongst individuals for all 11 genes (Fig 1A). The second exception was the gene ALB, which displayed high variance across all tissue types (Fig 1). With the possible exception of ALB, there were no systematic differences in gene expression levels between infected and uninfected individuals for any candidate RG or tissue type (Fig 1). For ALB, there was some evidence that uninfected individuals tended to have higher Ct values (lower expression) than infected individuals in lung (Fig 1B), GI2 (Fig 1D) and GI4 (Fig 1E) tissues.

### Candidate RG stability: GeNorm

**Fig 2:**
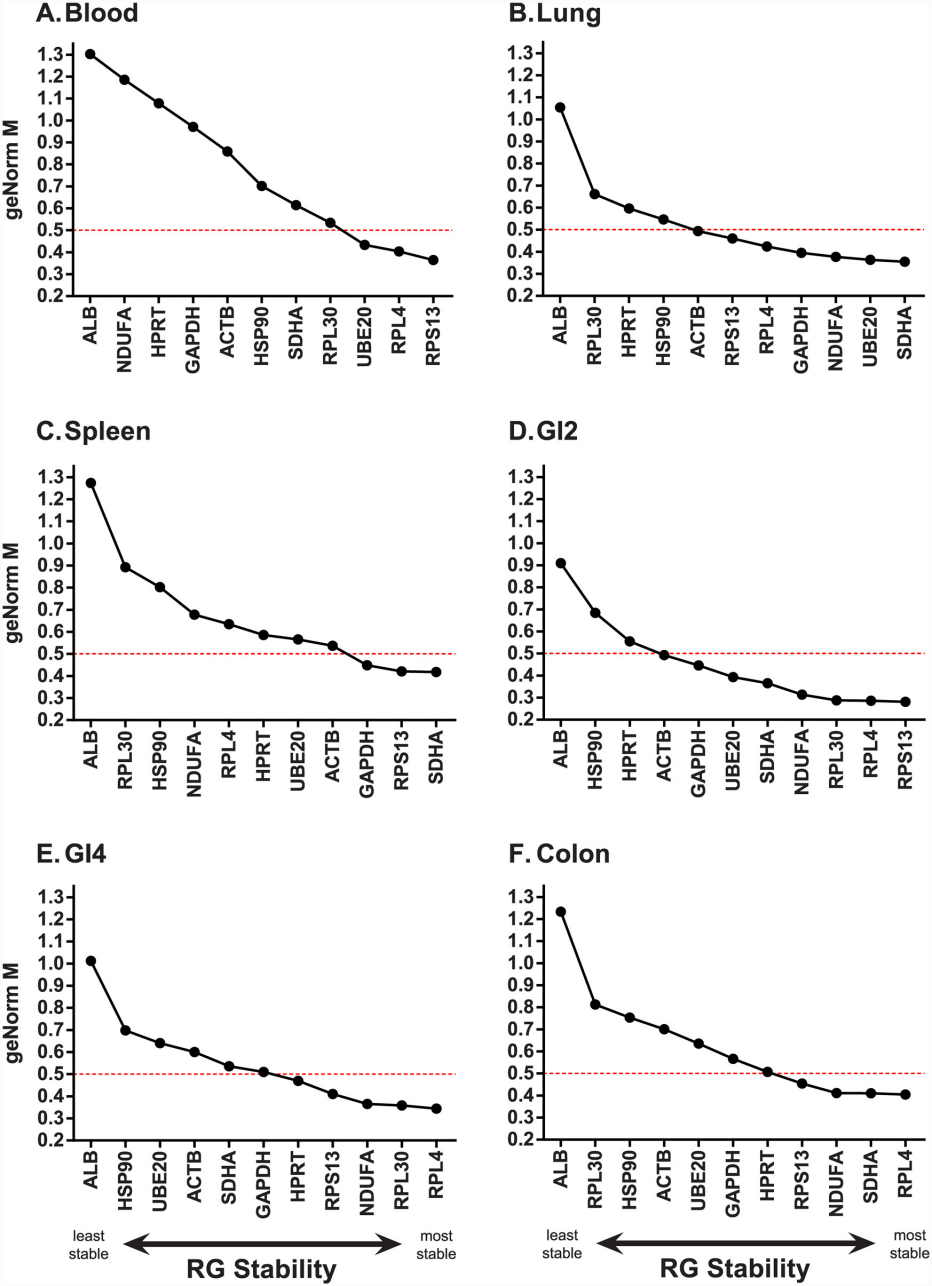
GeNorm stability rankings (M value) of eleven candidate reference genes amongst six mallard tissues. (A) Blood. (B) Spleen. (C) Liver. (D) GI2 (distal jejunum). (E) GI4 (distal ileum). (F) Colon. Data is plotted from least stable (left) to most stable (right) gene. Genes with an M value below 0.5 (red dashed line) are considered stable.

For all tissue types, ALB was consistently ranked the least stable gene (Fig 2, S2 Table). The rankings of all other candidate RGs were highly tissue dependant. For example, RPL4 displayed low stability in spleen (Fig 2C) but high stability in all other tissues (Fig 2). Similarly, NDUFA was not stable in blood or spleen (M value above 0.5) but was stable in the remaining tissues. The number of candidate RGs per tissue with acceptable stability (M value below 0.5) ranged from three (blood, spleen) to eight (GI2). With the exception of lung tissue, at least one of the three ribosomal protein genes (RPL4, RPL30 and/or RPS13) was consistently ranked amongst the most stable candidate RGs (S2 Table).

**Fig 3:**
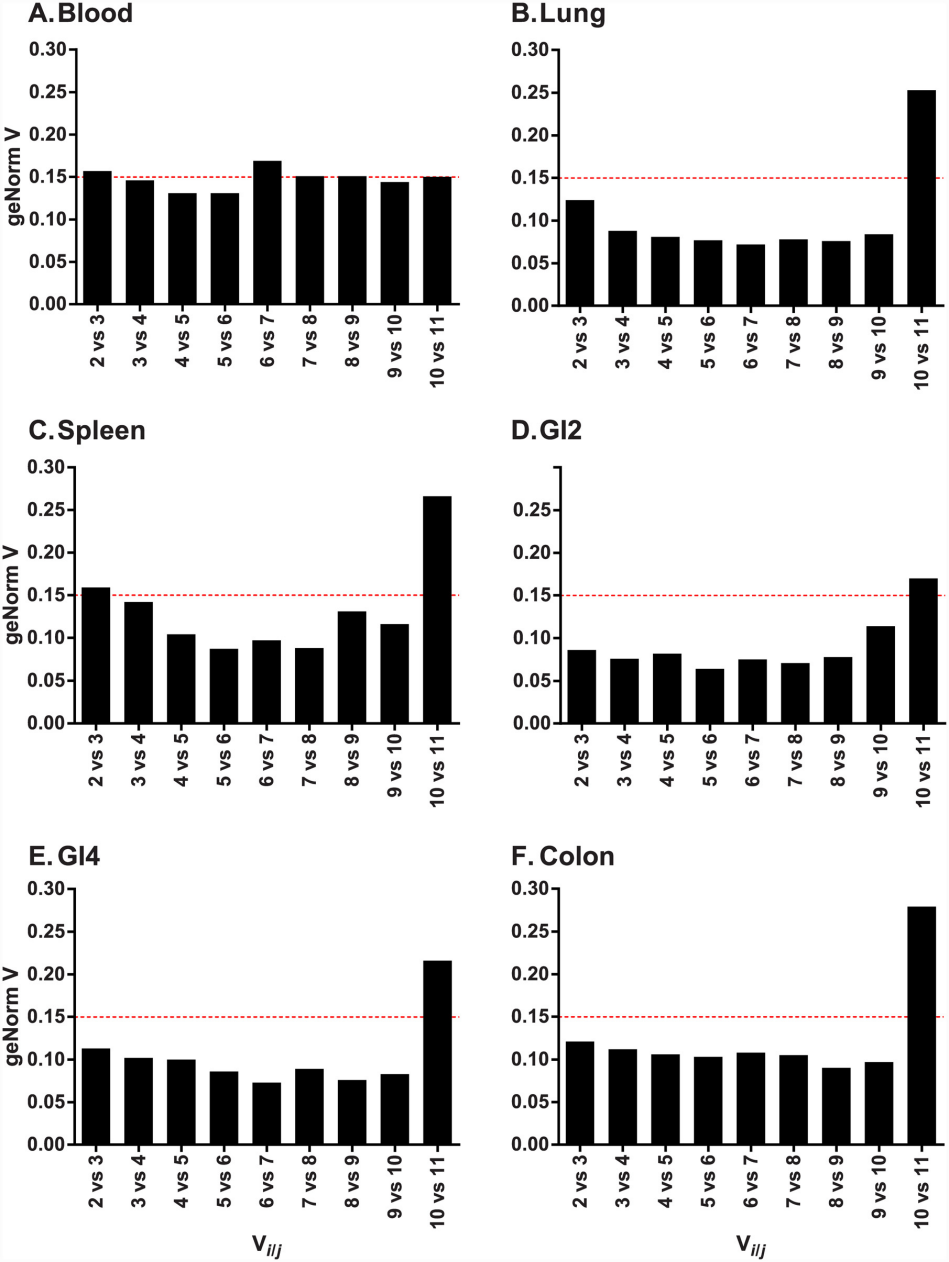
The number of RGs required for data normalisation. Y-axis represents the GeNorm V value and the X-axis is V_i/j_ where “*i*” is V calculated for *n* genes and “*j*” is *n* + 1 genes. If the V value for a given comparison of V_i/j_ falls below 0.15 (red dashed line), then the “*i*” number of genes is sufficient for normalisation.

To determine the number of RGs required for data normalisation, we used a cut-off V value of 0.15 or lower, as recommended in Vandesompele et al. [20]. For lung, GI2, GI4 and colon, we found that the combination of the two most stable candidate RGs was adequate to reach this threshold (Fig 3B, 3D–3F and Table 2), whereas for blood and spleen three RGs were required (Fig 3A and 3C). In general, the threshold of 0.15 was only strongly violated when ALB was included in the analysis (final bar in each graph in Fig 3), again highlighting its lack of suitability as an RG for our samples.

**Table 2:** Selected reference genes per tissue. Combined stability value for the best combination of genes as calculated in (A) GeNorm and (B) NormFinder. For GeNorm, the number selected was that required to reach a threshold stability V value of lower than 0.15; for NormFinder the recommended combination of the two best genes are provided. Note that stability values are not directly comparable between GeNorm and NormFinder, as each algorithm uses its own stability index.

	(A) GeNorm		(B) NormFinder	
	Best combination	Stability value	Best combination	Stability value
**Blood**	UBE20, RPL4 & RPS13	0.146	UBE20 & RPL4	0.121
**Lung**	UBE20 & SDHA	0.124	SDHA & RPS13	0.109
**Spleen**	RPS13, SDHA & GAPDH	0.142	RPL4 & RPS13	0.104
**GI2**	RPL4 & RPS13	0.086	GAPDH & NDUFA	0.099
**GI4**	RPL4 & RPL30	0.113	RPS13 & HPRT	0.119
**Colon**	SDHA & RPL4	0.121	SDHA & RPL4	0.034

### Candidate RG stability: NormFinder

**Fig 4:**
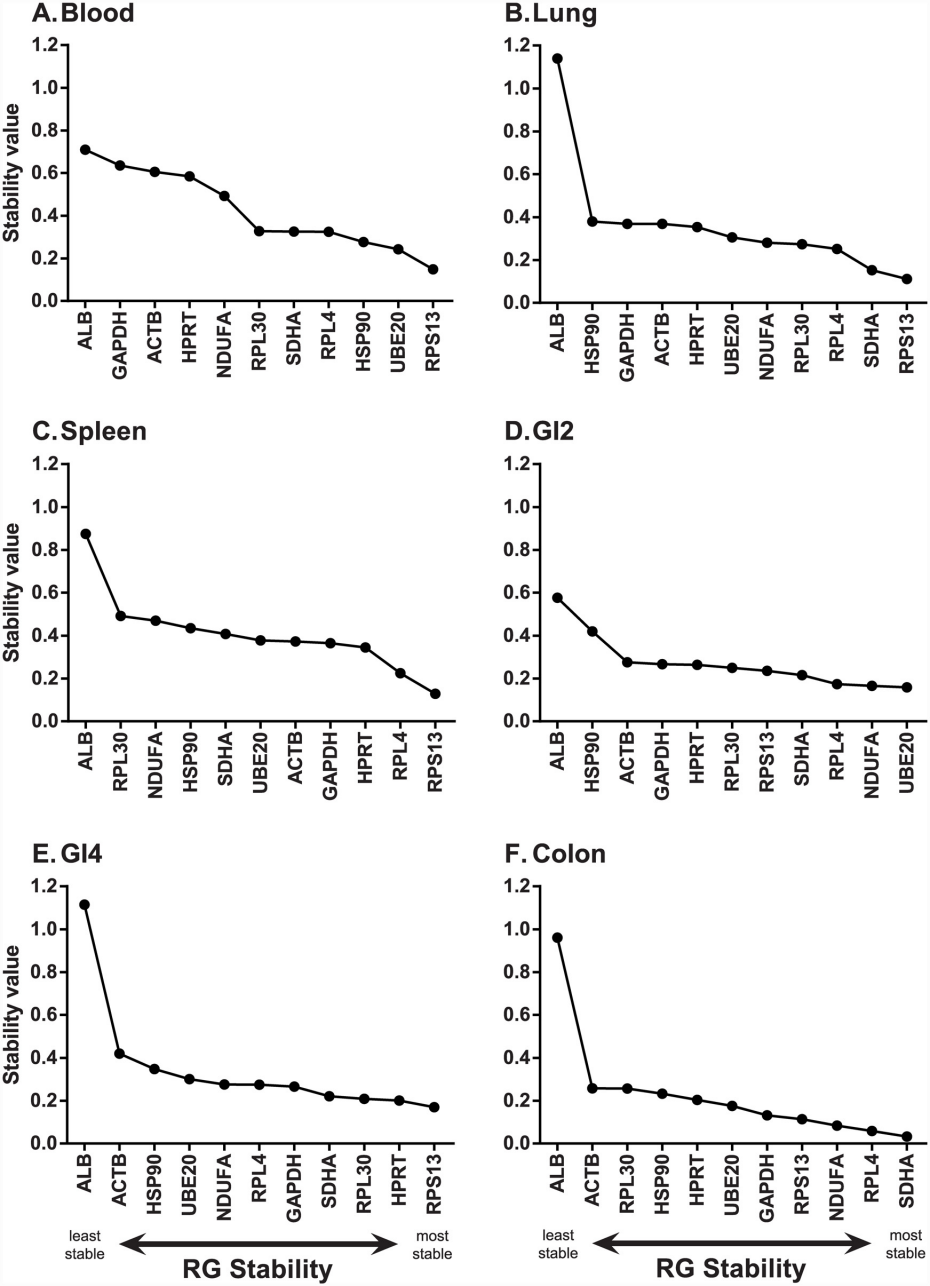
NormFinder stability rankings of eleven candidate reference genes amongst six mallard tissues. (A) Blood. (B) Spleen. (C) Liver. (D) GI2 (distal jejunum). (E) GI4 (distal ileum). (F) Colon. Data is plotted from least stable (left) to most stable (right) gene.

RGs belonging to the same regulatory pathway may be co-regulated and this can influence stability measures in GeNorm [20, 22, 31]. Our set of candidate RGs comprised three ribosomal proteins (RPL4, RPL30 and RPS13). In the GeNorm analysis, two ribosomal proteins were selected within the best combination of stable RGs in blood, GI2 and GI4. To test whether these genes were stable independent of the stability of the other, we additionally tested RG stability using the NormFinder algorithm. Additionally, the use of NormFinder allowed us to determine whether there were systematic differences in expression between infected and uninfected individuals by assigning samples as belonging to two treatment groups. We found that two ribosomal proteins were selected as the pair of most stable RGs in spleen (Table 2, Fig 4, S2 Table), but not for the other tissues. Indeed, only in colon was the exact same combination of two RGs (SDHA and RPL4) found to be the most stable by both algorithms (Table 2, Figs 2F & 4F, S2 Table). There was also good congruence between the programs for blood tissue, whereby both selected UBE20 and RPL4, however in the GeNorm analysis RPS13 was additionally selected in order to achieve a V value of below 0.15. For two further tissues (lung, spleen), both analyses agreed on one of the two most stable RGs, but differed in their selection of the other (Table 2, S2 Table). For example, in lung, both analyses selected SDHA as within the top two most stable RGs, but GeNorm paired this gene with UBE20 whereas NormFinder paired it with RPS13. With the exception of spleen, the pair of RGs selected by NormFinder were genes found to be stable in GeNorm (M value lower than 0.15, S2 Table). For spleen tissue, one of the RGs selected by NormFinder (RPL4) displayed low stability in GeNorm (M value 0.635).

## Discussion

GAPDH and ACTB are the most commonly chosen RGs in gene expression studies of vertebrates [18]. This is also true for studies of gene expressional changes due to infection in mallards, with these two genes overwhelmingly favoured as data normalisers (Table 3). However, we show here that both of these genes perform poorly as data normalisers for mallards across several different tissue types. ACTB was never selected as a suitable RG and was often ranked amongst the least stable (Figs 2 & 4), and its use has also been questioned in other contexts [18, 32]. GAPDH performed slightly better, being selected within the stable combination in spleen (GeNorm) or GI2 (NormFinder). However in most contexts, other genes had higher stability than GAPDH, and this gene was never selected by both algorithms in tandem. Notably, every previous study of disease-related gene expression in mallards has used a single RG to normalise data (Table 3). As stated in the MIQE guidelines [19], using a single RG to normalise qPCR data can bias results and is strongly discouraged (see also [18]). Use of qPCR to quantify gene expression without appropriate regard for experimental design, quality control and/or data interpretation can have profound yet often undetectable effects on conclusions drawn. Indeed, publication of the MIQE guidelines themselves were motivated by a spurious study linking autism to the measles mumps and rubella vaccine in children with inflammatory bowel disease [33, 34]. The fact that nearly all previous studies of mallard immune gene expression after infection are based on single, unvalidated RGs is therefore concerning. Furthermore, the one previous study to test RG stability in mallards concluded that 18s rRNA was suitable to use as a single data normaliser [17], although this gene was only compared to GAPDH and ACTB. We chose not to include 18S rRNA in our panel of RGs because it is a ribosomal RNA (rRNA) transcript. Pilot studies showed this gene to have much higher transcription, resulting in lower Ct values, than the mRNA candidate RGs (data not shown). Transcript levels (and therefore Ct values) between GOIs and RGs should be similar to ensure transcripts are subject to comparable kinetic interactions during qPCR [20], and few GOIs are expressed at the same level as 18S rRNA [35]. Additionally, rRNA and mRNA are transcribed by different RNA polymerases and thus regulatory changes in one may not affect the other [18, 36].

**Table 3:** Gene expression papers in Mallard and Pekin ducks. This summary is limited to those studies assessing mRNA transcriptional changes of genes of interest in response to infection of live animals with a pathogen followed by qPCR profiling of gene expression.

Pathogen	RG(s) used	Stability of RGs tested?	Reference
AIV	ACTB	No	[37]
AIV	ACTB	No	[38]
AIV	ACTB	No	[39]
AIV	ACTB or GAPDH*	No	[40]
AIV	GAPDH	No	[41]
AIV	GAPDH	No	[13]
AIV	GAPDH	No	[42]
AIV	GAPDH	No	[43]
AIV	GAPDH	No	[44]
AIV	GAPDH	No	[45]
AIV	GAPDH	No	[46]
AIV	GAPDH	No	[47]
AIV	GAPDH	No	[15]
AIV	GAPDH	No	[48]
AIV	18s rRNA	Yes^#^	[49]
AIV	18s rRNA	No	[50]
AIV	18s rRNA	Yes^†^	[51]
Duck hepatitis virus	ARBP	No	[52]
Duck hepatitis virus	ACTB	No	[14]
Duck hepatitis virus	ACTB	No	[53]
Duck hepatitis virus	ACTB	No	[54]
Duck hepatitis virus	GAPDH	No	[55]
Duck hepatitis virus	GAPDH	No	[56]
Duck hepatitis virus	GAPDH	No	[57]
Duck hepatitis virus	GAPDH	No	[58]
Newcastle disease virus	GAPDH	No	[59]
Newcastle disease virus	GAPDH	No	[60]
Duck Tembusu virus	ACTB	No	[61]
*Riemerella anatipestifer*	ACTB	No	[16]
*Escherichia coli*	ACTB	No	[62]

*Different RGs used in Pekin and Mallard.

^#^ Methods section states that stability of RGs was tested, but data not shown.

^†^ RG stability tested in a previous study by the same group.

Amongst the eleven putative mallard RGs tested, eight were selected as belonging to the stable RG pair for a given tissue at least once in our samples. The exceptions were ACTB, ALB and HSP90 –these genes were never selected as stable in any tissue for either analyses. Importantly, we found that different RGs were selected for different tissues, highlighting the importance of testing stability for every new tissue analysed. Within the GeNorm analyses, only RPS13 had a stability M value lower than 0.5 for every tissue tested (Fig 2), and this gene was also one of the most consistently stable across tissues via NormFinder analyses (Fig 4). One of the known biases with GeNorm is that it tends to rank co-regulated genes highly, independent of their individual expression stabilities, due to the pairwise comparison approach employed [20, 63, 64]. Given that our set of putative RGs included three ribosomal proteins, we additionally used NormFinder to test expression stability, also allowing us to compare uninfected with infected individuals explicitly. While both algorithms tended to rank ribosomal proteins highly, GeNorm selected ribosomal protein genes within the combination of 2–3 RGs required for stability more often (three times) than NormFinder (once). We thus could not rule out the possibility that there was some degree of co-regulation of ribosomal proteins.

One of the benefits of using GeNorm is that it provides an analysis of the number of RGs required for stabilisation whereas NormFinder does not; this is a crucial component of RG selection. For the samples tested here, the use of two RGs was sufficient for stabilisation (GeNorm V value lower than 0.15) for all tissues except blood and spleen, for which three RGs were required. However, for blood and spleen the V value threshold of 0.15 or lower was only marginally violated by the use of two RGs, suggesting that with a different sample set two RGs may be sufficient for these tissues also. Two RGs are considered the minimum that should be used for data normalisation [19, 20]. In general, the use of two RGs is favoured by researchers when feasible, because the use of additional normalisers can increase costs substantially.

Only two genes (ALB and HSP90) had GeNorm M values over 0.5 for every tissue tested, as such these genes cannot be considered RGs in the context of our study. In particular, HSP90 may be inappropriate for use as an RG in viral infection models because it acts as a chaperone of viral replication [65–67], but may be stable in non-pathological contexts [68]. The fact that ALB tended to have higher expression in infected than uninfected individuals in lung, GI2 and GI4 tissues (Fig 1) suggests that ALB may be upregulated in response to AIV infection in ducks. While albumin has not previously been shown to be associated with antiviral immune responses, it has been implicated in regulation of bacteria [69], yeast [69, 70] and eukaryotic parasites [71]. The remaining genes displayed adequate stability in at least a subset of analyses, and we recommend that, at a minimum, these nine genes are included in future panels when testing stability of putative RGs in mallards. It should be noted that pathogen infection is known to modulate gene expression [72, 73], therefore the stability/instability of candidate RGs in our study cannot easily be extrapolated to other treatment regimens. Inclusion of all the candidate RGs described here could therefore prove useful for studies of mallards subject to different experimental treatments. Furthermore, the candidate RGs described here provide a useful starting point for gene expression studies in other species of waterfowl, and indeed other avian taxa.

These results highlight the utility of testing RG stability via more than a single algorithm, followed by careful choice of the best RGs to use for data normalisation. There is no simple mathematical method with which to synthesise the results of different RG stability testing software, because they use different algorithms and require different input data (raw Ct values versus linearized data) and therefore cannot easily be compared [74]. The best approach is to choose RGs that are shown to be stable (and therefore score highly) across different algorithms [74]. The fact that our GeNorm and NormFinder analyses were not highly congruent might be due to the fact that NormFinder took into consideration the inter- and intra-group variation between infected and uninfected individuals, or could be due to the differences and associated with transforming raw Ct values into relative quantities employed by each algorithm.

## Conclusions

The use of single and/or non-stable RGs to normalise qPCR can lead to large and unpredictable errors in the estimation of gene expression (reviewed in [18, 75]). As such, the pool of potential RGs provided here offers a useful tool for normalising qPCR data when assessing gene expression in waterfowl, particularly mallards. We recommend future studies of gene expression in response to pathogen infection in ducks should first test the stability of the putative reference genes ACTB, GAPDH, HPRT, NDUFA, RPL4, RPL30, RPS13, SDHA and UBE20. Studies investigating non-pathological treatments could additionally consider the inclusion of HSP90 and ALB. Determining which genes are stable, and therefore appropriate for normalising data, within the context of the samples under consideration should be the crucial first step in all future studies of gene expression in mallards and other waterfowl.

## Supporting Information

S1 Appendix Additional materials and methods. (DOCX)

S1 Fig Experimental set-up. The experiment ran for 10 days from time point (*t*) -3 days before the start point until +7 days after the start point. Ducks (where *n* denotes the number of individuals) were housed in experimental rooms (black rectangles, where the length of the rectangle indicates the length of time ducks were housed in the room). Experimental procedures (red ellipses) took place at indicated (arrows) time points.
(TIF)

S1 Table Samples used in analyses. All available uninfected (control) samples were chosen (samples 1–5), as well as one third (*n* = 9) of the infected individuals (samples 6–33), spread across time points (0.5–7 days post infection, dpi, and inoculated ducks, innoc.). Different samples were chosen for each tissue as shown, where X denotes that the sample was selected for that tissue.
(DOCX)

S2 Table Stability values for each candidate reference gene per tissue. Stability values calculated in GeNorm and NormFinder (NormFind). The ranking of each individual gene in shown in brackets from most stable (1) to least stable (11) gene for that tissue. Genes shown in bold are those selected as most stable pair of RGs for the given analysis. Note that the most stable pair of RGs in NormFinder is not necessarily the two genes with the highest independent stability values (see also Table 2).
(DOCX)
