# Supplementary material for: A Panel of Stably Expressed Reference Genes for Real-Time qPCR Gene Expression Studies of Mallards (Anas platyrhynchos)
Source: PLoS One. 2016 Feb 17;11(2):e0149454. doi: 10.1371/journal.pone.0149454 (PMC4757037; doi:10.1371/journal.pone.0149454)
Supplement: S1 Appendix — (DOCX) [file pone.0149454.s001.docx]

**S1 Appendix: Additional Materials and Methods**

*RNA extraction*

Before RNA extraction, tissues were treated with RNA*later*®-ICE (Ambion, Austin, TX) following manufacturer’s guidelines. Briefly, 1.5 mL of pre-chilled (-80°C) RNA*later*®-ICE was added to each tube of tissue. Tubes were flicked to ensure tissue pieces were floating freely in solution, and then stored at -20°C for 16 – 24 hours. RNA was extracted using the RiboPure Kit (Ambion) following the manufacturers protocol. Briefly, approximately 70 mg tissue (range 40-100 mg) was removed from RNA*later*®-ICE solution, blotted on lint-free tissue to remove excess liquid, then placed in a RINO^®^ (Next Advance, Averill Park, NY ) 1.5 mL tube containing three 3.2mm stainless tell beads and 1 mL of TRI Reagent (Ambion). The sample was homogenised in a Bullet Blender® Storm 24 (Next Advance) on speed setting 10 for 4 minutes, with extra time added in 30 second increments if required. The homogenate was incubated at room temperature for 5 minutes, centrifuged (4°C) at 12,000 x g for 10 min and 1mL of supernatant placed in a fresh 1.5 mL tube. 100 μL of bromochloropropane (BCP) was added to the homogenate, vortexed and incubated for 5 minutes before centrifugation (4°C) at 12,000 x g for 10 min. 400 μL of the aqueous phase was placed in a new tube and mixed with 200 μL of 100% EtOH. This was then loaded onto a filter cartridge and washed twice with wash solution before final elution in 100 µl of elution buffer. Extracted RNA was quantified (NanoDrop 2000, Thermo Scientific, Wilmington, DE) and stored at 4°C (short-term) or –80°C (long-term).

*DNase treatment and quality control*

To ensure that contaminating DNA was removed from RNA extracts, they were passed through a DNase treatment. In short, 50 μL reactions containing 200 ng/μL of total

extract were treated using the Turbo DNA-free kit (Ambion) following

manufacturer’s instructions. DNase treated RNA samples were then re-quantified, and stored

at -80ºC until required. The quality and integrity of treated RNA samples was visually inspected by loading on a 1% agarose gel containing 2% bleach, following the protocol provided in [[1](#_ENREF_1)]. Bands for rRNA and tRNA were clearly visible, whereas a high molecular weight band representing genomic DNA (gDNA) was not present (S2 Fig). To further confirm absence of gDNA, a random selection of eight samples of treated RNA from each tissue extract were subjected to real-time PCR amplification using primers anchored in introns. In all cases, no amplification was observed with intronic primers (data not shown).


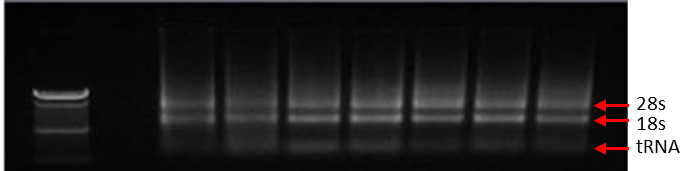


**S2 Fig. RNA bleach gel**. DNase treated RNA extracts were run on a 1% agarose gel containing 2% bleach. Bands for large (28S) and small (18S) subunit rRNA and tRNA are clearly visible, whereas a large molecular weight gDNA band is absent.

*cDNA synthesis*

DNase treated RNA was diluted to a tissue-specific standardised concentration, and used as template to synthesise cDNA. The concentrations used were as follows: blood 27.8 ng/µl, spleen 62.5 ng/µl, lung 56.5 ng/µl, GI2 106.2 ng/µl, GI4 78.5 ng/µl and colon 72.2 ng/µl. Briefly, for each sample, 11 μL diluted RNA, 400 ng of random hexamers (Qiagen, Valencia, CA) and 10 mM dNTPs (Qiagen) were heated to 65ºC for five minutes and subsequently cooled down on ice for one minute. 200 U of SuperScript III reverse transcriptase (Invitrogen, Carlsbad, CA), 1X first strand buffer (250 mM Tris-HCl [pH 8.3], 375 mM KCl and 15 mM MgCl2), 0.1 M dithiothreitol (DTT) and 40 U RNasin Plus (Promega, Madison, WI) were added to a final volume of 20 μl. cDNA was synthesised with the following incubation profile: 25ºC for five min, 50ºC for 60 min, 70ºC for 15 min and a final cooling to 4ºC. cDNA was stored at -20ºC until use. Negative controls comprising all of the above components except the reverse transcriptase enzyme (RT-minus reactions) were performed for every tissue. For all tissues except GI2, there was no amplification of RT-minus controls. For GI2 samples, a small amount of amplification occurred, however Cq values were always above 38 and the melt peak was a lower temperature, hence this amplification was clearly distinguishable from the expected amplification product (data not shown).

*Confirmation of individual AIV infection status*

Oropharyngeal and faecal swabs were taken before the start of the experiment, and daily from every individual during the experiment (until euthanized) to confirm AIV infection status via real-time reverse transcriptase PCR (rRT-PCR). Briefly, samples were collected using sterile cotton tipped applicators, placed in virus transported media (Hank's balanced salt solution containing 0.5% lactalbumin, 10% glycerol, 200 U/mL penicillin, 200 µg/mL streptomycin, 100 U/mL polymyxin B sulfate, 250 µg/mL gentamicin, and 50 U/mL nystatin (Sigma, St Loius, MO) and frozen to -80°C within 4 hours of collection. When required for analysis, samples were thawed, diluted 1:4 with PBS and RNA extracted using the Viral NA Large Volume Kit and MagNA Pure 96 robot (Roche, Mannheim, Germany). Thereafter, the One Step Real-Time PCR Kit (Qiagen, Hilden, Germany) was used to amplify a short fragment of the AIV matrix gene by the use of the Light Cycler480 (Roche), as previously described [[2](#_ENREF_2),[3](#_ENREF_3)]. Cq values under 40 were considered positive. Additionally, NP-ELISA was used to ensure that ducks had no antibodies indicating previous influenza infection at the prior to the start of the experiment, as described in Tolf *et al*. [[4](#_ENREF_4)]. Briefly, 0.8 mL of blood from each individual was centrifuged to separate the sera, which was subsequently screened for the presence of anti-NP antibodies using the FlockCheck Avian Influenza Virus Antibody Test Kit, (IDEXX, Hoofddorp, Netherlands). Results were interpreted following the manufacturers’ recommendations, where a sample to negative control ratio (S/N) value below 0.5 indicated a positive result. Using these methods, all individuals were confirmed AIV negative at the start of the experiment, and control ducks remained AIV negative throughout. Experimentally- and contact-infected ducks were rapidly infected with AIV and remained AIV positive throughout the experiment (data not shown).

**Additional References**

1. Aranda PS, LaJoie DM, Jorcyk CL (2012) Bleach gel: A simple agarose gel for analyzing RNA quality. Electrophoresis 33: 366-369.

2. Spackman E, Senne DA, Myers TJ, Bulaga LL, Garber LP, et al. (2002) Development of a real-time reverse transcriptase PCR assay for type A influenza virus and the avian H5 and H7 hemagglutinin subtypes. J Clin Microbiol 40: 3256-3260.

3. Wille M, Avril A, Tolf C, Schager A, Larsson S, et al. (2015) Temporal dynamics, diversity, and interplay in three components of the virodiversity of a Mallard population: Influenza A virus, avian paramyxovirus and avian coronavirus. Infect Genet Evol 29: 129-137.

4. Tolf C, Latorre-Margalef N, Wille M, Bengtsson D, Gunnarsson G, et al. (2013) Individual variation in influenza A virus infection histories and long-term immune responses in Mallards. PLoS One 8: e61201.
