# Supplementary figures and images for: A Panel of Stably Expressed Reference Genes for Real-Time qPCR Gene Expression Studies of Mallards (Anas platyrhynchos)
Source: PLoS One. 2016 Feb 17;11(2):e0149454. doi: 10.1371/journal.pone.0149454 (PMC4757037; doi:10.1371/journal.pone.0149454)

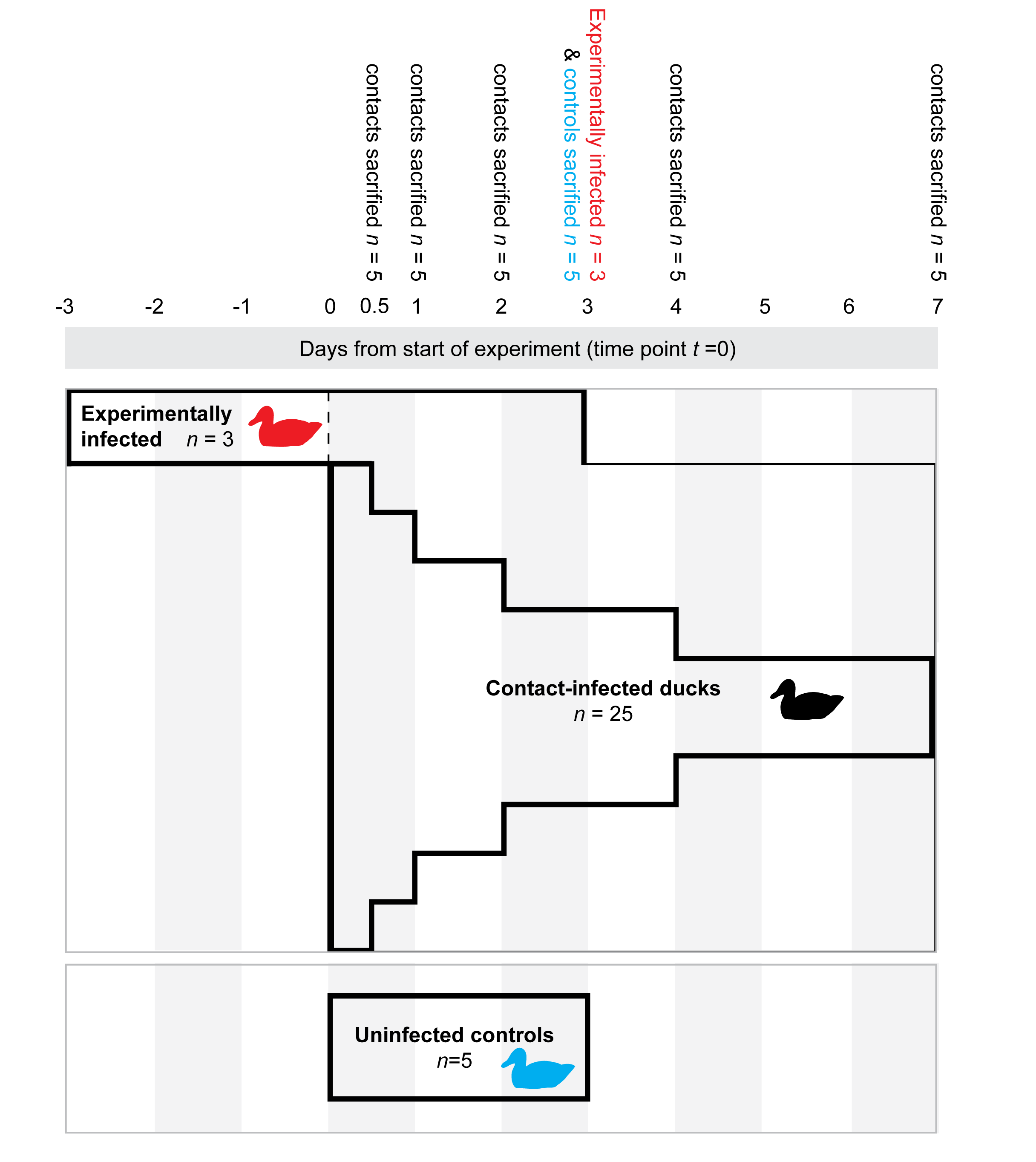

Supplement: S1 Fig — The experiment ran for 10 days from time point (t) -3 days before the start point until +7 days after the start point. Ducks (where n denotes the number of individuals) were housed in experimental rooms (black rectangles, where the length of the rectangle indicates the length of time ducks were housed in the room). Experimental procedures (red ellipses) took place at indicated (arrows) time points. (TIF) [file pone.0149454.s002.tif]
