# Supplementary material for: A Panel of Stably Expressed Reference Genes for Real-Time qPCR Gene Expression Studies of Mallards (Anas platyrhynchos)
Source: PLoS One. 2016 Feb 17;11(2):e0149454. doi: 10.1371/journal.pone.0149454 (PMC4757037; doi:10.1371/journal.pone.0149454)
Supplement: S1 Table — All available uninfected (control) samples were chosen (samples 1–5), as well as one third (n = 9) of the infected individuals (samples 6–33), spread across time points (0.5–7 days post infection, dpi, and inoculated ducks, innoc.). Different samples were chosen for each tissue as shown, where X denotes that the sample was selected for that tissue. (DOCX) [file pone.0149454.s003.docx]

**S1 Table. Samples used in analyses.** All available uninfected (control) samples were chosen (samples 1 - 5), as well as one third (*n* = 9) of the infected individuals (samples 6 - 33), spread across time points (0.5 – 7 days post infection, dpi, and inoculated ducks, innoc.). Different samples were chosen for each tissue as shown, where X denotes that the sample was selected for that tissue.

| **Time point** | **Control** | | | | | **0.5 dpi** | | | | | **1 dpi** | | | | | **2 dpi** | | | | | **4 dpi** | | | | | **7 dpi** | | | | | **Innoc.** | | |
| --- | --- | --- | --- | --- | --- | --- | --- | --- | --- | --- | --- | --- | --- | --- | --- | --- | --- | --- | --- | --- | --- | --- | --- | --- | --- | --- | --- | --- | --- | --- | --- | --- | --- |
| **Sample** | **1** | **2** | **3** | **4** | **5** | **6** | **7** | **8** | **9** | **10** | **11** | **12** | **13** | **14** | **15** | **16** | **17** | **18** | **19** | **20** | **21** | **22** | **23** | **24** | **25** | **26** | **27** | **28** | **29** | **30** | **31** | **32** | **33** |
| **Blood** | X | X | X | X | X |  | X |  |  | X |  |  |  | X |  | X |  |  | X |  | X |  | X |  |  | X |  |  |  |  |  | X |  |
| **Lung** | X | X | X | X | X |  |  |  |  | X |  |  | X | X |  |  | X |  |  | X |  | X |  |  |  |  |  | X | X |  |  | X |  |
| **Spleen** | X | X | X | X | X |  | X |  |  |  | X |  |  | X |  | X |  | X |  |  | X |  | X |  |  |  |  |  | X |  |  |  | X |
| **GI2** | X | X |  | X | X | X |  |  |  |  |  | X | X |  |  | X |  |  | X |  | X | X |  |  |  |  |  |  | X |  | X |  |  |
| **GI4** |  | X | X | X | X |  | X |  |  |  |  | X |  | X |  |  | X | X |  |  | X | X |  |  |  |  |  |  |  | X |  | X |  |
| **Colon** | X | X | X | X | X | X |  |  |  |  |  | X |  | X |  | X |  | X |  |  | X |  |  | X |  |  |  |  |  | X |  | X |  |
