# Supplementary material for: A Panel of Stably Expressed Reference Genes for Real-Time qPCR Gene Expression Studies of Mallards (Anas platyrhynchos)
Source: PLoS One. 2016 Feb 17;11(2):e0149454. doi: 10.1371/journal.pone.0149454 (PMC4757037; doi:10.1371/journal.pone.0149454)
Supplement: S2 Table — Stability values calculated in GeNorm and NormFinder (NormFind). The ranking of each individual gene in shown in brackets from most stable (1) to least stable (11) gene for that tissue. Genes shown in bold are those selected as most stable pair of RGs for the given analysis. Note that the most stable pair of RGs in NormFinder is not necessarily the two genes with the highest independent stability values (see also Table 2). (DOCX) [file pone.0149454.s004.docx]

**S2 Table. Stability values for each candidate reference gene per tissue**. Stability values calculated in GeNorm and NormFinder (NormFind). The ranking of each individual gene in shown in brackets from most stable (1) to least stable (11) gene for that tissue. Genes shown in bold are those selected as most stable pair of RGs for the given analysis^.^ Note that the most stable pair of RGs in NormFinder is not necessarily the two genes with the highest independent stability values (see also Table 2).

|  | **Blood** | | **Spleen** | | **Lung** | | **GI2** | | **GI4** | | **Colon** | |
| --- | --- | --- | --- | --- | --- | --- | --- | --- | --- | --- | --- | --- |
| **Gene name** | **GeNorm** | **NormFind** | **GeNorm** | **NormFind** | **GeNorm** | **NormFind** | **GeNorm** | **NormFind** | **GeNorm** | **NormFind** | **GeNorm** | **NormFind** |
| ACTB | 0.859 (7) | 0.606 (9) | 0.537 (4) | 0.373 (5) | 0.494 (7) | 0.369 (8) | 0.493 (8) | 0.276 (9) | 0.600 (8) | 0.420 (10) | 0.701 (8) | 0.258 (10) |
| ALB | 1.303 (11) | 0.710 (11) | 1.274 (11) | 0.875 (11) | 1.054 (11) | 1.140 (11) | 0.910 (11) | 0.577 (11) | 1.012 (11) | 1.115 (11) | 1.234 (11) | 0.961 (11) |
| GAPDH | 0.971 (8) | 0.636 (10) | **0. 449 (3)** | 0.365 (4) | 0.395 (4) | 0.369 (9) | 0.446 (7) | **0.173 (8)** | 0.510 (6) | 0.266 (5) | 0.567 (6) | 0.132 (5) |
| HPRT | 1.078 (9) | 0.585 (8) | 0.586 (6) | 0.345 (3) | 0.596 (9) | 0.354 (7) | 0.555 (9) | 0.264 (7) | 0.470 (5) | **0.201 (2)** | 0.507 (5) | 0.204 (7) |
| HSP90 | 0.0.702 (6) | 0.277 (3) | 0.802 (9) | 0.435 (8) | 0.546 (8) | 0.380 (10) | 0.684 (10) | 0.420 (10) | 0.698 (10) | 0.348 (9) | 0.753 (9) | 0.233 (8) |
| NDUFA | 1.186 (10) | 0.493 (7) | 0.678 (8) | 0.470 (9) | 0.377 (3) | 0.281 (5) | 0.314 (4) | **0.165 (2)** | 0.365 (3) | 0.276 (7) | 0.411 (3) | 0.084 (3) |
| RPL4 | **0.404 (2)** | **0.325 (4)** | 0.635 (7) | **0.225 (2)** | 0.424 (5) | 0.252 (3) | **0.286 (2)** | 0.174 (3) | **0.344 (1)** | 0.275 (6) | **0.405 (1)** | **0.059 (2)** |
| RPL30 | 0.534 (4) | 0.328 (6) | 0.892 (10) | 0.492 (10) | 0.661 (10) | 0.274 (4) | 0. 288 (3) | 0.250 (6) | **0.359 (2)** | 0.209 (3) | 0.813 (10) | 0.257 (9) |
| RPS13 | **0.364 (1)** | 0.149 (12) | **0.421 (2)** | **0.225 (1)** | 0.460 (6) | **0.153 (1)** | **0.281 (1)** | 0.236 (5) | 0.410 (4) | **0.170 (1)** | 0.454 (4) | 0.132 (4) |
| SDHA | 0.614 (5) | 0.326 (5) | **0. 418 (1)** | 0.408 (7) | **0.355 (1)** | **0.153 (2)** | 0.365 (5) | 0.216 (4) | 0.536 (7) | 0.266 (4) | **0.410 (2)** | **0.032 (1)** |
| UBE20 | **0.433 (3)** | **0.243 (2)** | 0.566 (5) | 0.378 (6) | **0.363 (2)** | 0.306 (6) | 0.393 (6) | 0.159 (1) | 0.640 (9) | 0.300 (8) | 0.636 (7) | 0.176 (6) |
